# Direct-Acting Antiviral Treatment of HCV Infection Does Not Resolve the Dysfunction of Circulating CD8^+^ T-Cells in Advanced Liver Disease

**DOI:** 10.3389/fimmu.2019.01926

**Published:** 2019-08-13

**Authors:** Agatha Vranjkovic, Felicia Deonarine, Shaima Kaka, Jonathan B. Angel, Curtis L. Cooper, Angela M. Crawley

**Affiliations:** ^1^Chronic Disease Program, Ottawa Hospital Research Institute, Ottawa, ON, Canada; ^2^Department of Biochemistry, Microbiology and Immunology, University of Ottawa, Ottawa, ON, Canada; ^3^Division of Infectious Diseases, The Ottawa Hospital, Ottawa, ON, Canada; ^4^School of Epidemiology and Public Health, University of Ottawa, Ottawa, ON, Canada; ^5^Clinical Epidemiology Program, Ottawa Hospital Research Institute, Ottawa, ON, Canada; ^6^Department of Biology, Carleton University, Ottawa, ON, Canada

**Keywords:** CD8+ T-cells, HCV, liver, direct-acting antivirals, fibrosis

## Abstract

Chronic hepatitis C virus (HCV) infection disrupts immune functions, including that of cytotoxic CD8^+^ T-cells which are important mediators of immune response. While HCV cure aims to eliminate long term sequelae of infection, whether direct-acting antiviral (DAA) treatment results in immune reconstitution remains unclear. We and others have reported generalized CD8^+^ T-cell dysfunction in chronic HCV infection and our research suggests that the degree of liver damage is a factor in this process. Our recent research indicates that liver fibrosis is not readily reversed after DAA-mediated clearance of chronic HCV infection. We therefore examined the function of circulating CD8^+^ T-cell subsets in chronic HCV infection in the context of liver fibrosis severity, determined by ultrasound elastography and Metavir F-score system. We observed progressive shifts in CD8^+^ T-cell subset distribution in HCV-infected individuals with advanced liver fibrosis (F4) compared to minimal fibrosis (F0-1) or uninfected controls, and this remained unchanged after viral cure. Impaired CD8^+^ T-cell function was observed as a reduced proportion of CD107^+^ and perforin^+^ late effector memory cells in HCV^+^(F4) and HCV^+^(F0-1) individuals, respectively. In HCV^+^(F4) individuals, nearly all CD8^+^ T-cell subsets had an elevated proportion of perforin^+^ cells while naïve cells had increased proportions of IFN-γ^+^ and CD107^+^ cells. These exaggerated CD8^+^ T-cell activities were not resolved when evaluated 24 weeks after completion of DAA therapy and HCV clearance. This was further supported by sustained, high levels of cell proliferation and cytolytic activity. Furthermore, DAA therapy had no effect on elevated concentrations of systemic inflammatory cytokines and decreased levels of inhibitory TGF-β in the plasma of HCV^+^(F4) individuals, suggesting HCV infection and advanced liver disease result in a long-lasting immune activating microenvironment. These data demonstrate that in chronic HCV infection, liver fibrosis severity is associated with generalized hyperfunctional CD8^+^ T-cells, particularly with perforin production and cytotoxicity, and this persists after viral clearance. Whether DAA therapy will eliminate other related long-term sequelae in HCV^+^(F4) individuals remains an important research question.

## Introduction

One third of circulating immune cells pass through the liver every minute, wherein host responses and tolerance are delicately balanced. Hepatic and extra-hepatic changes in liver disease are associated with immune dysfunction (depression/exhaustion, overstimulation/activation) and systemic inflammation, depending on the liver disease severity and etiology (1, 2). Over 50 million people worldwide are chronically-infected with hepatitis C virus (HCV), of which ≈15–30% will silently develop advanced liver fibrosis/cirrhosis, symptomatic liver disease, and predisposing to end-stage liver disease and portal hypertension (3, 4) with high mortality rates. Even after HCV cure, regression of liver fibrosis is slow, if it occurs at all, as reported after treatment with the former gold-standard IFN-α + ribavirin therapy (5, 6) and more recently with DAA therapy (7). Despite the treatment of HCV infection with either of those therapies, individuals with advanced liver disease are still at risk of developing hepatocellular carcinoma (HCC) (8, 9) and many remain on liver transplant lists (10). The functions of many innate and adaptive immune system cells are impaired in chronic HCV infection, particularly HCV-specific CD8^+^ T-cells (11–16), while underlying mechanisms remain to be understood. Chronic HCV infection may have a more extensive effect on *all* CD8^+^ T-cells, both those directed to HCV and other antigens, as markers of exhaustion are observed on CD8^+^ T-cells in the blood, spleen and liver (17–20). We have observed significant impairment of cytokine signaling and survival of the entire CD8^+^ T-cell compartment in the blood and liver in HCV infection and this was associated with the severity of liver disease (21). Whether the functional capacity of circulating CD8^+^ T-cells in HCV infection with advanced liver disease is markedly different than with minimal liver fibrosis is not known. Given the importance of CD8+ T-cells in responses to viruses, intracellular bacteria and parasites and tumor cell surveillance, all of which remain a challenge to positive outcomes post-DAA therapy, investigation of this perspective is required. Also, whether DAA therapy can restore immunological dysfunction remains unclear.

Infection with HCV is typically cleared with 8–12 weeks of direct-acting antiviral (DAA) therapy in most individuals (>98%), although HCV genotype (genotype 3) and liver fibrosis stage (F4) are associated with reduced treatment efficiency. While new DAA therapies have achieved spectacular results for viral clearance, the immunorestorative effects of DAA therapy are not known. Restoration of innate immune cell function with DAA therapy, such as NK cells in a study comprised of 42% cirrhotic individuals (22) are countered by reports of how mucosal-associated invariant T (MAIT) cells are not reinvigorated with DAA therapy in cirrhotic individuals (23). Similarly, there are conflicting reports on reversibility of impaired HCV-specific CD8^+^ T-cell function after viral clearance with 48 weeks of IFN-α + ribavirin, suggesting irreversible immune cell dysfunction (11, 24). Detection of impaired HCV-specific CD8^+^ T-cells in chronic HCV infection prior to antiviral therapy predisposes re-infected individuals to developing chronic HCV infection yet again (25). Studies have demonstrated the restoration of HCV-specific T-cells after DAA therapy yet have not isolated effects of liver fibrosis severity. It remains to be determined whether the generalized immune dysfunction observed in chronic HCV infection persists long after DAA therapy.

Understanding the immunological changes associated with advanced liver fibrosis in chronic HCV infection is imperative to overcome the remaining relevant clinical outcomes after therapy as many patients will not experience a reversal of liver fibrosis for many years, if at all. Our recent studies have confirmed that liver fibrosis reversal occurs slowly, if at all, 1 year after the completion of DAA therapy in HCV-infected individuals alongside persisting liver steatosis and inflammation, as measured by controlled attenuation parameter scores (the highest of which were among those with advanced liver fibrosis) [Doyle et al. (26)]. Since HCV is the only chronic viral infection that can currently be cured, there is a unique opportunity to investigate whether immune dysfunction is reversed with viral clearance following successful therapy. An understanding of the functional potential of the entire CD8^+^ T-cell compartment may offer insights on future responses to remaining clinical sequalae post-cure (e.g., other infections, re-infection with new strains of HCV, vaccination efficacy, and cancer surveillance to prevent HCC and extrahepatic cancers). In this study, we sought to determine if liver disease severity is associated with generalized dysfunction of CD8^+^ T-cells in chronic HCV mono-infection before and after DAA therapy. We evaluated CD8^+^ T-cell phenotypic distribution and various indicators of immune and cytolytic function in circulating CD8^+^ T-cells. We demonstrate for the first time that advanced liver fibrosis/cirrhosis is associated with generalized immune dysfunction that persists long after HCV cure.

## Methods

### Study Subjects

This study was carried out in accordance with the recommendations of the guidelines established by the Ottawa Health Science Network Research Ethics Board with written informed consent from all subjects. All subjects gave written informed consent in accordance with the Declaration of Helsinki. The protocol was approved by the Ottawa Health Science Network Research Ethics Board. Staff in The Ottawa Hospital Clinical Investigations Unit consented the study participants and collected blood samples. Study groups included healthy HCV^−^ individuals (*n* = 9) and chronically-infected HCV^+^ (>6 months HCV RNA^+^) treatment naïve individuals (see Table 1 for patient characteristics). HCV-infected individuals were classified based on the degree of liver fibrosis. Liver fibrosis evaluation was performed within 6 months of blood sample collection using transient elastography. This score, in kiloPascals (kPa) was converted to the METAVIR staging system (F0-F4). The HCV-infected individuals were grouped as follows: HCV^+^(F0-1) (≤ 7.0 kPa, *n* = 11) or HCV^+^(F4) (≥12.5 kPa, *n* = 8). The latter group was restricted to individuals with Child-Pugh class A liver disease. Individuals with Child-Pugh B and C cirrhosis (i.e., decompensated liver disease) were excluded from this study. The average age of controls was 43.6 ± 10.1, which was lower than HCV^+^ study groups (F0-1: 57.3 ± 7.8 yrs *p* = 0.002 and F4: 59.9 ± 11.7 *p* = 0.001). There was no significant difference in the age of the HCV^+^ groups.

**Table 1:** Clinical characteristics of control and HCV^+^ study groups.

Parameter	Chronic HCV (F4^a^)	Chronic HCV (F0-1^a^)	Uninfected controls
Number of participants	7	11	9
Sex	6 (M), 1 (F)	7 (M), 4 (F)	4 (M), 5 (F)
Mean age years ± SD (range)	59.9 ± 11.7 (45–76)	57.3 ± 7.8 (41–64)	43.6 ± 10.0 (25–54)
Race	Caucasian (6), East Indian (1)	Caucasian (10), First Nations (1)	Caucasian (9)
Mean baseline fibrosis score (kPa^b^) ±SD (24 weeks post-SVR^c^)	22.5 ± 14.94 (10.5 ± 2.6, *n =* 4)	5.4 ±1.7 (5.9 ±1.8, *n =* 4)	n/a
IgG anti-CMV^d^	3/4 seropositive	4/4 seropositive	n/a

a *Liver fibrosis score (Metavir fibrosis/cirrhosis F4, ≥12.5 kPa); (Metavir minimal fibrosis F0-1, ≤7.0 kPa)*.

b *kPa, KiloPascal (determined by transient elastography)*.

c *SVR, sustained virological response (undetectable HCV RNA 12 weeks after treatment cessation)*.

d *CMV, cytomegalovirus, assessed in subjects treated with DAA therapy. n/a, Not assessed. SD, standard deviation*.

To evaluate the effects of DAA therapy on CD8^+^ T-cell function in this context, HCV-infected individuals about to undergo HCV treatment were grouped based on liver fibrosis stage prior to DAA treatment initiation (see Table 1 for patient characteristics). These individuals were treated with a 12 week regimen of paritaprevir (protease inhibitor), ombitasvir (NS5a inhibitor), dasabuvir (non-nucleoside polymerase inhibitor) with or without ribavirin (Abbott Laboratories, Chicago, IL, USA). Blood samples used for this study were collected at day 0 and 24 weeks post-SVR treatment. All treated individuals studied here achieved a sustained virological response, SVR (i.e., undetectable HCV RNA by 12 weeks after treatment cessation). Seropositivity for cytomegalovirus (CMV) was determined by ELISA (MP Biomedicals, Solon, Ohio, USA). There was no significant difference in the average age of individuals in the HCV^+^ groups receiving DAA therapy (F0-1: 51.8 ± 9.0, F4: 67.3 ± 9.5). The effect of age on cell function could not be evaluated in this data set as there were not young vs. older individuals to compare within these groups.

### Cell Isolation and Culture

Blood samples were collected in heparin-containing tubes and PBMCs were isolated by Ficoll gradient centrifugation and frozen for later use following published methods (27). After an overnight incubation of thawed PBMCs, CD8^+^ T-cells were isolated using the EasySep™ Human CD8 Positive Selection Kit II (STEMCELL Technologies, Vancouver, British Columbia, Canada). Cells (2 × 10^6^ cells/ml) were cultured in plates pre-coated overnight at 4°C with anti-CD3 (10 μg/mL, kindly provided by Dr. S.H. Lee, University of Ottawa) and soluble anti-CD28 antibodies (10 μg/mL, Biolegend, San Diego, CA, USA). Cells were cultured for 48 h before analysis of cell functions, as determined by time course and dose response experiments (Figure S1).

### Cell Subset Determination

Subsets of CD8^+^ T-cells were distinguished on the basis of cell surface receptor expression as described previously (28), using antibodies specific for CD45RA-ECD (clone 2H4LDH11LDB9, Beckman Coulter), CCR7-APC/CY7 (Clone G043H7, BioLegend, San Diego, CA, USA), and CD27-PC5 (clone 1A4CD27, Beckman Coulter. The following subsets were analyzed in this study: Naïve (CD45RA^+^CCR7^+^CD27^+/−^), Effector (E, CD45RA^+^CCR7^−^CD27^+/−^), Early Effector Memory (e-EM, CD45RA^−^CCR7^−^CD27^+^), Late Effector Memory (l-EM^−^, CD45RA^+/−^CCR7^−^CD27^−^), and Central Memory (CM, CD45RA^−^CCR7^+^CD27^+/−^). Cell subsets were assessed by flow cytometry using a FC500 machine (Beckman Coulter, Marseille, France). This began by gating on the lymphocyte population based on FSC/SSC, followed by doublet-exclusion and live/dead staining using the Live-Dead Fixable Stain Kit (Molecular Probes, Eugene, Oregon, USA). Cells were evaluated by applying the fluorescence minus-one color compensation strategy followed by gating on CD45RA^+^ and CD45RA^−^ subsets, followed by subset determination based on CCR7 and CD27 expression on the latter subsets. Data were analyzed using FlowJo software (FLOWJO, LLC, Ashland, Oregon).

### Assessment of CD8^+^ T-Cell Functions

#### Proportion of IFN-γ^±^ Cells

Six hours prior to the end of the 48 h anti-CD3/-CD28 cell stimulation, cells were treated with Brefeldin A (15 μg/mL, Millipore SIGMA, Oakville, ON, Canada). After labeling cells with subset determination antibodies and cell fixation (see above), cells were permeabilized with saponin (Millipore SIGMA) in 10% Human AB serum (Vally Biomedical Inc., Winchester, VA, USA) + anti-human IFN-γ-FITC antibodies (clone 4S.B3, BioLegend) or IgG1κ-FITC isotype control (clone MOPC-21, BioLegend) and evaluated by flow cytometry.

#### Proportion of Perforin^±^ Cells

After cell subset labeling and fixation, cells were labeled for perforin using anti-human perforin-FITC antibodies (clone δG9, BD Pharmingen, BD Bioscience, San Jose, CA, USA) or IgG2b- FITC isotype control antibodies (clone 27–35, BD Pharmingen, BD Bioscience). To minimize non-specific binding, human AB serum (10%) was included in all buffers.

#### Proportion of Degranulating (CD107a^±^) Cells

Six hours before the end of the 48 h cell stimulation, cells were treated with monensin (8 μM, BD Bioscience) while incubating cells with anti-human CD107a-FITC antibody or IgG1κ-FITC (clones H4A3, and MOPC-21, respectively, BD Biosciences). Cells were then labeled with antibodies for cell subset determination, fixed and analyzed by flow cytometry.

### Plasma Cytokine Quantification

The concentrations of pro- and anti-inflammatory cytokines in plasma were quantified using multiplexing immunobead assays analyzed using the BioRad Luminex machine (Bio-Rad Laboratories, Hercules, CA, USA). Transforming growth factor β (TFG-β)−1,−2, and−3 were quantified using the TGF-Beta 1,2,3 Magnetic Bead Kit (Milliplex, Millipore SIGMA) while all remaining cytokines were assayed using the 48-plex Bio-Plex Pro™ Human Cytokine Screening Panel (BioRad, Mississauga, ON, CANADA).

### Data Analysis

Data were analyzed by either Student's one-way *t*-test, one-way ANOVA with Dunnett's post-test (*p* ≤ 0.05) or Mann-Whitney U-test with interquartile ranges (25 and 75%) with adjusted *p*-values, as appropriate. GraphPad Prism 5.0 software, Microsoft Excel and R programming language were used for statistical analyses and to plot data.

## Results

### Increased CD8^+^ Effector and Late Effector Memory T-cell Subsets in Untreated HCV Infection With Advanced Liver Fibrosis

**Figure 1:**
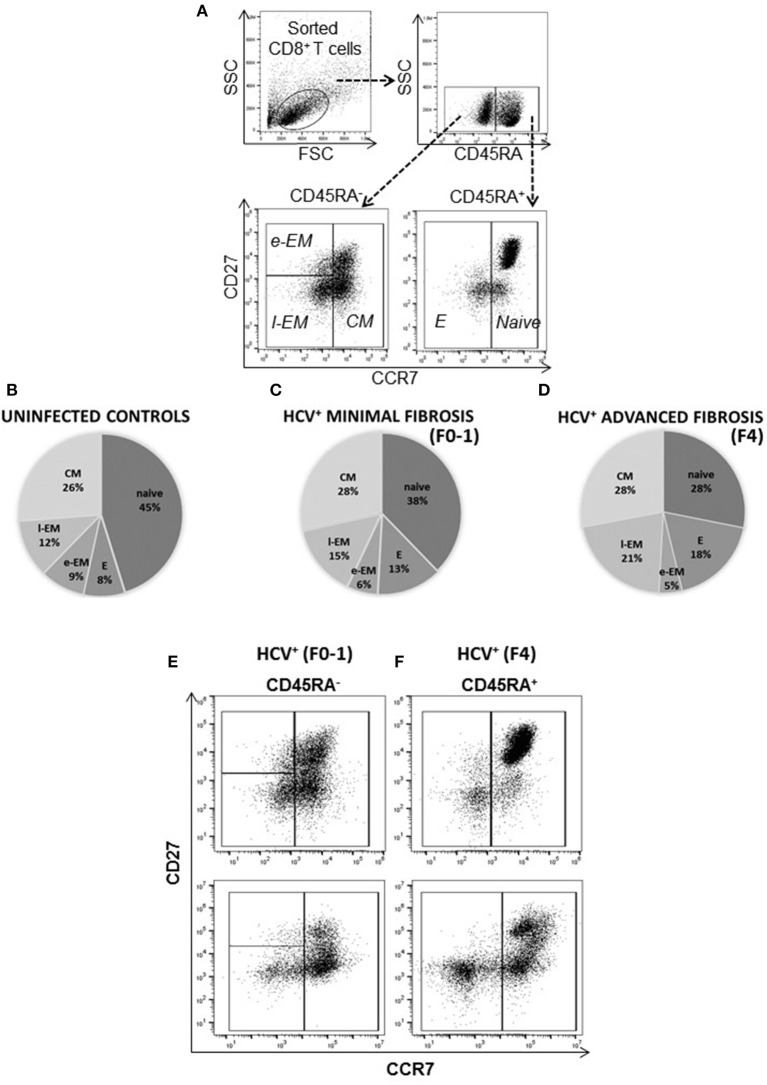
The degree of liver fibrosis in chronic HCV infection is associated with altered circulating CD8^+^ T-cell subset distribution. CD8^+^ T-cells were isolated from PBMCs for analysis of subset distribution. The proportions of cell CD8^+^ T-cell subsets were distinguished based on surface marker expression by flow cytometry as follows: Naïve (CD45RA^+^CCR7^+^CD27^+/−^), Effector (E, CD45RA^−^CCR7^−^CD27^−^), Early Effector Memory (e-EM, CD45RA^−^CCR7^−^CD27^+^), Late Effector Memory (l-EM, CD45RA^+/−^CCR7^−^CD27^−^), and Central Memory (CM, CD45RA^−^CCR7^+^CD27^+/−^). **(A)** Representative dot plots of the lymphocyte gate (forward vs. side scatter) and phenotype strategy are shown. The distribution of **(B)** uninfected controls (*n* = 9) and treatment naïve HCV^+^ individuals with **(C)** minimal (Metavir score F0-1, liver thickness ≤7.0 kPa, *n* = 9) or **(D)** advanced liver fibrosis/cirrhosis (F4, ≥12.5 kPa, *n* = 5) are shown with pie-charts [mean percentages (%), subset S.D. < ±10%]. Representative dot plots of CD8^+^ T-cell subset distribution in the **(E)** HCV^+^ (F0-1) and **(F)** HCV^+^ (F4) study groups are shown.

The distribution of blood-derived, CD8^+^ T-cell subsets was evaluated in HCV uninfected individuals (controls) and in untreated HCV^+^(F0-1) and HCV^+^(F4) individuals to detect any differences associated with liver fibrosis severity. Subset distribution in unstimulated cells was determined after 48 h of cell culture, alongside other cell function assays described herein. A time- and dose-dependent analysis of anti-CD3/CD28-stimulated CD8^+^ T-cells responses indicated responses after 6 h and additional responses after 48 h (Figure S1). The latter time point was selected as a means to reliably detect either increases or decreases in function and is consistent with approaches used in our previous work (21, 29). There was also a small but measurable *in vitro* stimulation effect on cell phenotype during this time. The proportion of naïve cells increased (10% more than unstimulated cells) while the proportion of e-EM and l-EM cells decreased (<5%) in controls and HCV^+^(F0-1) individuals. Similar effects were observed in cells from HCV^+^(F4) individuals as well as decreases in the proportion of E and CM cells (<5%) (Figure S2). There were no significant differences in the distribution of any CD8^+^ T-cell subset between controls and HCV^+^(F0-1) individuals, as determined by one-way ANOVA and Dunnett's post-test (Figure 1). In HCV^+^(F4) individuals, there was no difference in the proportion of CD8^+^ e-EM and CM CD8^+^ T-cells compared to controls. However, HCV^+^(F4) individuals had ~50% fewer naïve CD8^+^ T-cells (mean 27.47% ± 7.0 S.E., *n* = 7) than controls (mean 45.40% ± 5.4 S.E., *n* = 9), (ANOVA *p* = 0.03). This study group also had more than twice as many E cells (mean 17.7% ± 3.1 S.E.) and l-EM cells (mean 20.62% ± 6.4 S.E.) compared to controls (E mean 8.18% ± 4.6 S.E., l-EM mean 11.54% ± 1.5 S.E.) (ANOVA *p* = 0.05 and 0.002, respectively). Mean values of naïve and l-EM subsets in the HCV^+^(F4) group were lower than those of HCV^+^(F0-1) individuals, although this did not reach statistical difference. While the HCV^+^ individuals were significantly older than controls, we did not associate differences in subset proportions with age.

### More IFN-γ^+^ Circulating Naïve CD8^+^ T-cells in Untreated HCV Infection With Advanced Liver Fibrosis

**Figure 2:**
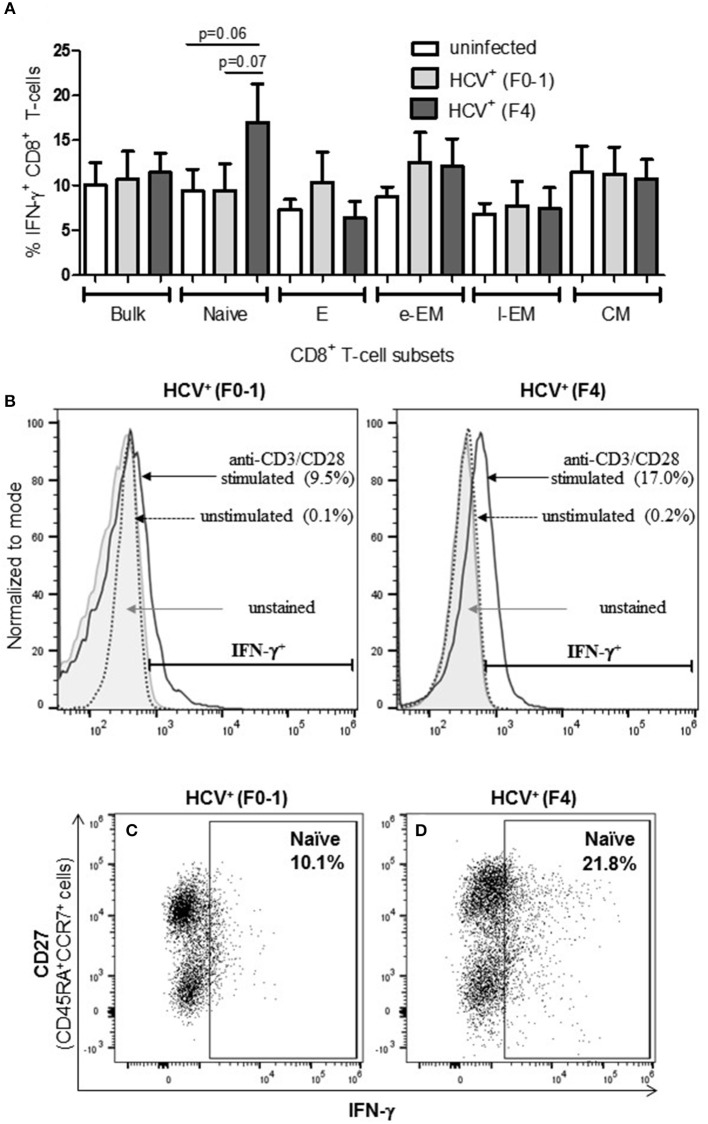
Advanced liver fibrosis/cirrhosis is associated with increased IFN-γ^+^ circulating naïve CD8^+^ T-cells in chronic HCV infection. After isolating CD8^+^ T-cells from PBMCs of uninfected controls (*n* = 9), HCV^+^ individuals with minimal or advanced liver fibrosis (F0-1, ≤7.0 kPa, *n* = 9; F4, ≥12.5 kPa, *n* = 5, respectively), cells were stimulated with anti-CD3/CD28 antibodies for 48 h, the proportion (%) of cells expressing intracellular IFN-γ was assessed by flow cytometry. **(A)** These data are summarized in bar graphs. **(B)** Histograms of IFN-γ flow cytometry traces of naïve cells to provide representative examples from each HCV^+^ group to show marker placement strategy relative to unstained cells and cells cultured with medium alone (Ctl) or stimulated with anti-CD3/CD28 antibodies and differences in %IFN-γ^+^ cells. The y-axis represents the relative number of cells after normalization to the mode (i.e., unit distribution) using FlowJo software. Representative dot plots of IFN-γ expression in naïve CD8^+^ T-cells in cells isolated from individuals with either **(C)** minimal or **(D)** advanced liver fibrosis are shown. Statistical trends in altered proportion of IFN-γ^+^ cells between groups are shown with a line and *p*-values approaching statistical significance (*p* ≤ 0.05) are shown, as determined by one-way ANOVA and Dunnett's post-test.

To determine if the production of the immune modulating cytokine IFN-γ by circulating CD8^+^ T-cells is associated with the severity of liver disease in chronic HCV infection, intracellular IFN-γ expression was assessed in anti-CD3/CD28-stimulated cells. Following stimulation of blood CD8^+^ T-cells, the proportion (%) of IFN-γ^+^ cells did not differ in any cell subset between the groups (Figure 2A). In stimulated cells from HCV^+^(F4) individuals, the proportion of IFN-γ^+^ naïve CD8^+^ T-cells (mean 16.99%) demonstrated a trend of a nearly 2-fold increase compared to controls and HCV^+^(F0-1) individuals (mean = 9.39% and 9.50%, respectively: one-way ANOVA and Dunnett's post-test, *p* = 0.06 and 0.07, respectively, Figures 2A–D).

### Increased Degranulation of Naïve Cells in Advanced Liver Fibrosis While l-EM Cells Were Reduced in This Capacity

**Figure 3:**
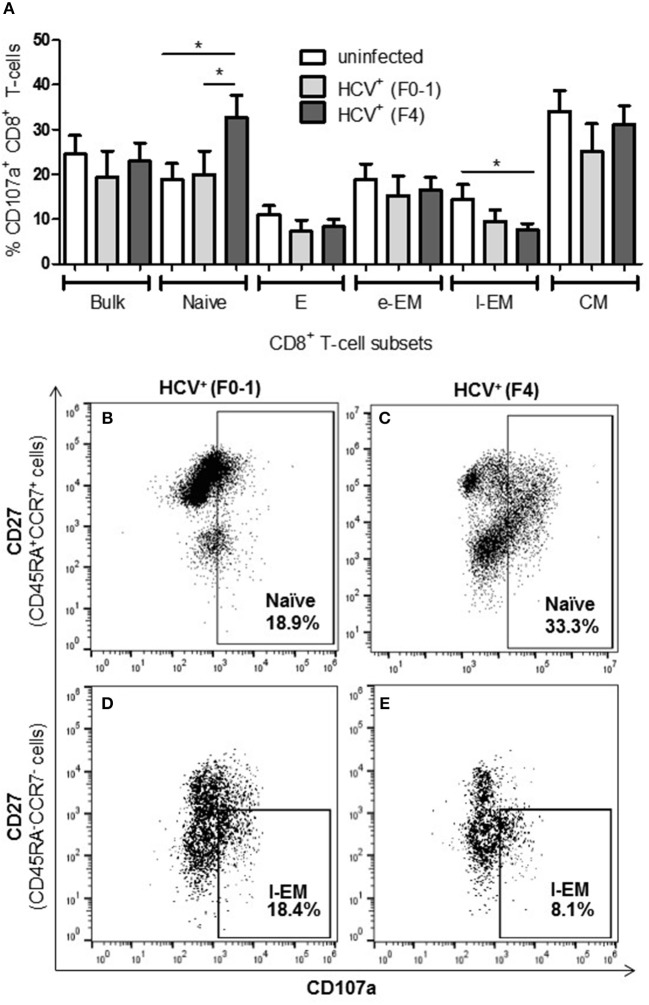
Evidence of increased degranulation is observed in circulating naïve CD8^+^ T-cells in HCV-infected individuals with advanced liver fibrosis while l-EM cells reduced their capacity to degranulate. The detection of CD107a, a marker of cell degranulation, was conducted by flow cytometry on anti-CD3/-CD28-stimulated CD8^+^ T-cells isolated from the blood of health controls and HCV^+^ individuals with minimal or advanced liver fibrosis. **(A)** The proportion of CD107a^+^ cells are summarized in a bar graph, by cell subset. Statistically significant changes are demonstrated with representative dot plots to compare CD107a expression in HCV-infected individuals with either minimal or advanced liver fibrosis in **(B,C)** naïve and **(D,E)** l-EM cell subsets. Statistically significant changes are indicated with a “^*^” (one-way ANOVA and Dunnett's post-test, *p* ≤ 0.05).

To further examine if altered CD8^+^ T-cell activity in HCV infection is associated with the degree of liver fibrosis, the detection of degranulation marker CD107a was evaluated following cell stimulation. The expression of CD107a is undetectable in unstimulated CD8^+^ T-cells, while anti-CD3/CD28 stimulation induces the exposure of this inner leaflet receptor, indicating the capacity of the cell to degranulate. There were no differences in the CD107a expression induced in CD8^+^ T-cells between any of the study groups (Figure 3A). There were significantly more CD107a^+^ naïve cells from HCV^+^(F4) individuals compared to controls or HCV^+^(F0-1) individuals (ANOVA *p* = 0.02 and 0.05, respectively, Figures 3A,B). There was also a significant decrease in the induced proportion of CD107a^+^ l-EM cells in HCV^+^(F4) individuals compared to controls (*p* = 0.04, ANOVA, Figures 3D,E), while no significant change was detected cells from HCV^+^(F0-1) individuals compared to the other groups. There were significantly more CD107a^+^ naïve cells in the HCV^+^(F4) group (Figure 3C) compared to the HCV^+^(F0-1) (Figure 3B) and control groups (*p* < 0.05, ANOVA).

### Increased Proportion of Perforin^+^ CD8^+^ T-cells in Untreated HCV Infection With Advanced Fibrosis

**Figure 4:**
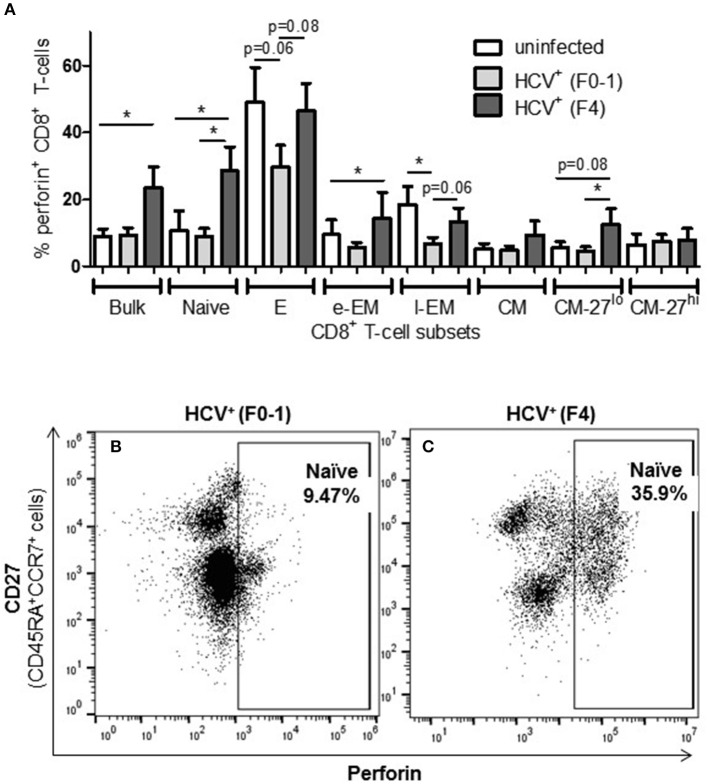
The proportion of perforin^+^ CD8^+^ T-cells induced following stimulation is increased in nearly all cell subsets in HCV-infected individuals with advanced liver fibrosis. After cell stimulation, the intracellular expression of perforin was measured by flow cytometry across several CD8^+^ T-cell subsets. **(A)** The data for perforin expression in cells from healthy controls and HCV-infected individuals with minimal or advanced liver fibrosis are summarized in a bar graph. **(B,C)** An example of a significant difference in cell subset expression of perforin between minimal and low fibrosis is shown with representative dot plots of naïve cells. Statistically significant changes are indicated with a “^*^” (one-way ANOVA and Dunnett's post-test, *p* ≤ 0.05) and statistical trends are indicated where *p*-values approach statistical significance.

To complement the CD107a degranulation observations above, expression of the lytic protein perforin was evaluated. Firstly, all study groups expressed a similar baseline level of perforin^+^ cells (≈5%). Following anti-CD3/-CD28 stimulation, the proportion of perforin^+^ CD8^+^ T-cells cells from HCV^+^(F4) individuals with advanced liver fibrosis (mean 23.39%) was significantly greater than HCV^+^(F0-1) individuals (mean 9.44%) or healthy controls (mean 9.0%) (*p* = 0.02, ANOVA) (Figure 4). This was most evident in naïve cells (mean 28.56% vs. HCV^+^(F0-1) 9.01%, *p* = 0.003 or vs. controls 10.70%, *p* = 0.04, Figures 4B,C). Subdividing CD8^+^ T-cell subsets based on the degree of CD27 expression has previously been shown to reveal diametrically opposed levels of perforin expression such that more CD27^lo^ cells express perforin compared to CD27^hi^ cells (30). In our data, this was most readily testable in the CM population due to the significant number of cells in each of the CD27^hi^ and CD27^lo^ subsets. We detected a significant increase in perforin^+^ cells in CD27^lo^ CM cells from HCV^+^(F4) individuals compared to HCV^+^(F0-1) individuals (*p* = 0.03, ANOVA). No such increase was observed in CD27^hi^ CM cells. Significantly more CD27^−^ naïve cells expressed perforin in the HCV^+^(F0-1) and (F4) groups compared to controls (*p* = 0.03 and = 0.02, respectively). An equivalent analysis of CD27^hi/lo^ cells in the l-EM subset was not informative due to a lack of significant cell numbers in the CD27^−^ subset.

A significant impairment in the proportion of induced perforin^+^ cells was observed in HCV^+^(F0-1) individuals, specifically in the E and l-EM subsets compared to controls (*p* = 0.06 and 0.02, respectively, ANOVA) whereas the proportion of perforin^+^ cells in those subsets in cells from HCV^+^(F4) individuals were nearly equivalent to that of controls. In HCV^+^(F4) individuals, E and naïve cells had the highest proportion of perforin^+^ cells among subsets, whereas far fewer naïve cells were perforin^+^ in the HCV^+^(F0-1) individuals and controls (*p* < 0.05, ANOVA).

### Direct-Acting Antiviral Therapy Does Not Normalize CD8^+^ T-cell Function in Advanced Liver Fibrosis

**Figure 5:**
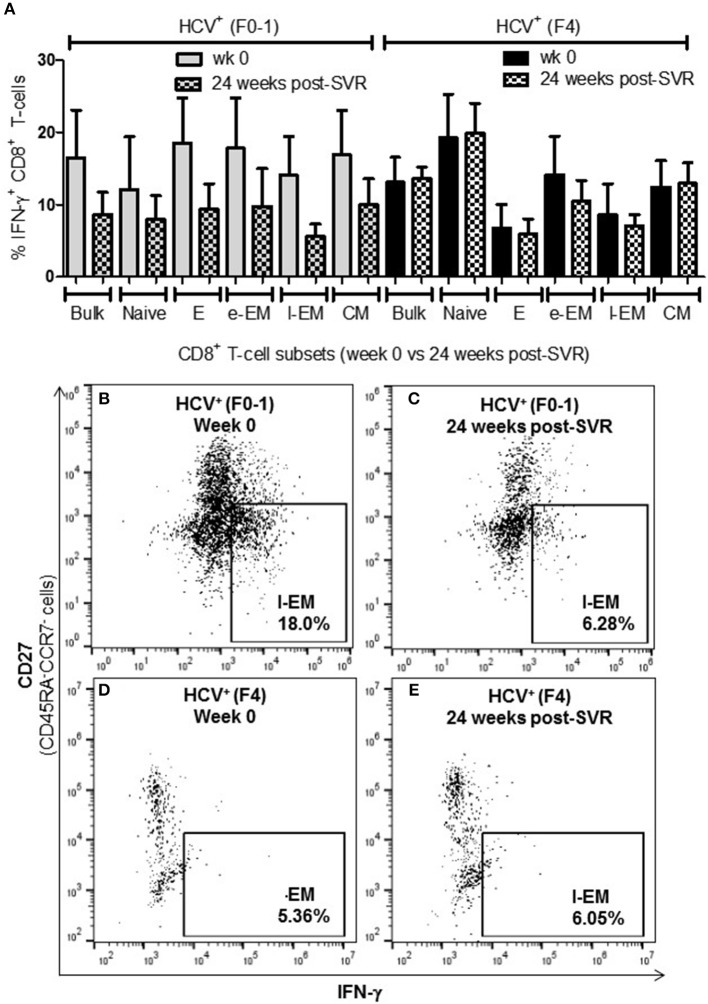
The proportion of IFN-γ^+^ CD8^+^ T-cells tended to decrease after DAA therapy in HCV-infected individuals with minimal fibrosis yet was sustained in a state of advanced liver fibrosis. Treatment-naïve HCV-infected individuals were treated with direct-acting anti-HCV (DAA) therapy for 12 weeks, and PBMCs were collected at wk 0 and 24 weeks post-SVR. Isolated CD8^+^ T-cells from PBMCs of HCV^+^ individuals with minimal or advanced liver fibrosis (F0-1, ≤7.0 kPa, *n* = 4; F4, ≥12.5 kPa, *n* = 4) when DAA treatment was initiated were stimulated with anti-CD3/-CD28 for 48 h and CD8^+^ T-cell functions were assessed. **(A)** A bar graph summarizes the proportion of IFN-γ^+^ CD8^+^ T-cells in HCV^+^ individuals with minimal or advanced liver fibrosis, before and after DAA therapy **(B–E)** Representative dot plots demonstrate the expression of IFN-γ in l-EM cells.

To determine whether the increased activity of CD8^+^ T-cells observed in chronic HCV infection is reversed following elimination of the virus with DAA therapy, the above functions were evaluated before the initiation of DAA therapy and 24 weeks post-SVR. While there was individual variation in the cellular responses after DAA therapy, CD8^+^ T-cells from HCV^+^(F0-1) individuals tended to have reduced functions by 24 weeks post-SVR. This was particularly evident in the proportion of IFN-γ^+^ and CD107a^+^ cells in all subsets, in which group means at 24 weeks post-SVR were typically 50% lower than week 0 (Figures 5A,B vs. C and 6A,B vs. C), although this did not reach statistical significance, perhaps due to the individual variation in responses and small group size. In the HCV^+^(F0-1) group, there were proportionally fewer perforin^+^ cells compared to the HCV^+^(F4) group, with levels often equivalent to unstimulated cells (Figure 7A). These low proportions of perforin^+^ cells did not change by 24 weeks post-SVR (e.g., Figures 7B vs. C). Therefore, HCV^+^(F0-1) individuals produced fewer perforin^+^ cells than HCV^+^(F4) individuals and had an overall tendency to reduce IFN-γ and CD107a responses after DAA therapy.

**Figure 6:**
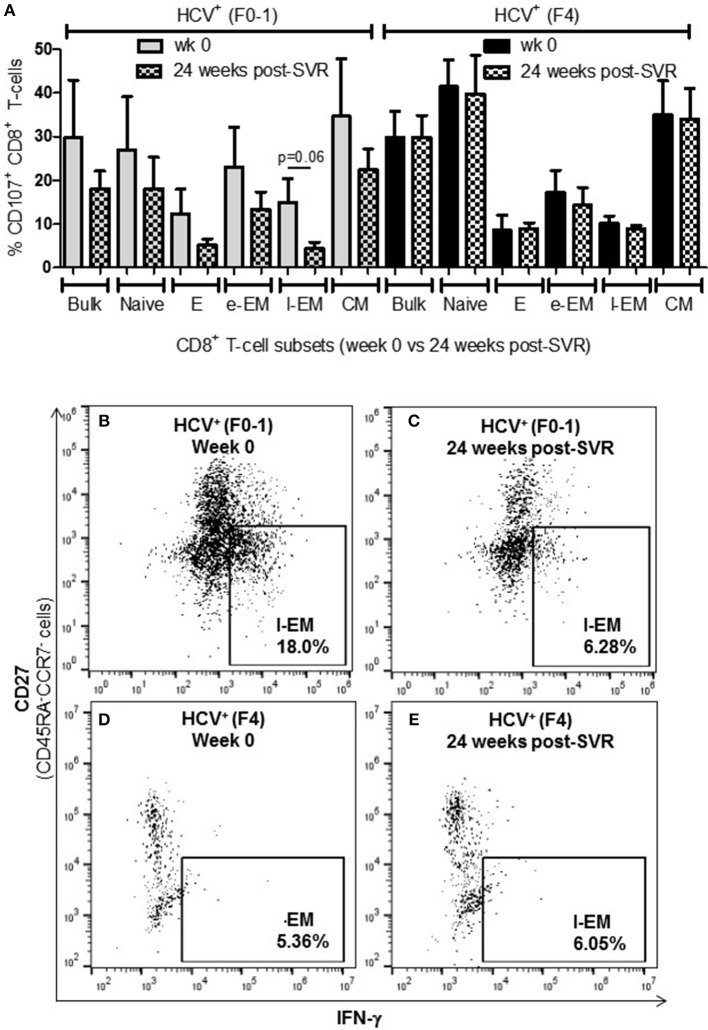
Levels of cell degranulation marker expression are sustained after HCV cure in individuals with advanced liver fibrosis. The presence of CD107a was evaluated in *in vitro* stimulated CD8^+^ T-cells isolated from HIV-infected individuals before treatment with direct-acting anti-HCV therapy (DAA) and 24 weeks post-SVR. **(A)** These data are summarized in a bar graph showing data for individuals with minimal liver fibrosis on the left and advanced fibrosis on the right (*n* = 4 in each study group). Data for week 0 data are shown in solid colored bars while data for 24 weeks post-SVR are shown in hatched bars. **(B,C)** Representative dot plots demonstrate the reduction in detectable CD107a^+^ l-EM cells from week 0 to 24 weeks post-SVR in an individual with minimal fibrosis. **(D,E)** Additional representative dot plots contrast the latter with sustained CD107a levels in l-EM cells of an individual with advanced liver fibrosis before and after DAA therapy.

**Figure 7:**
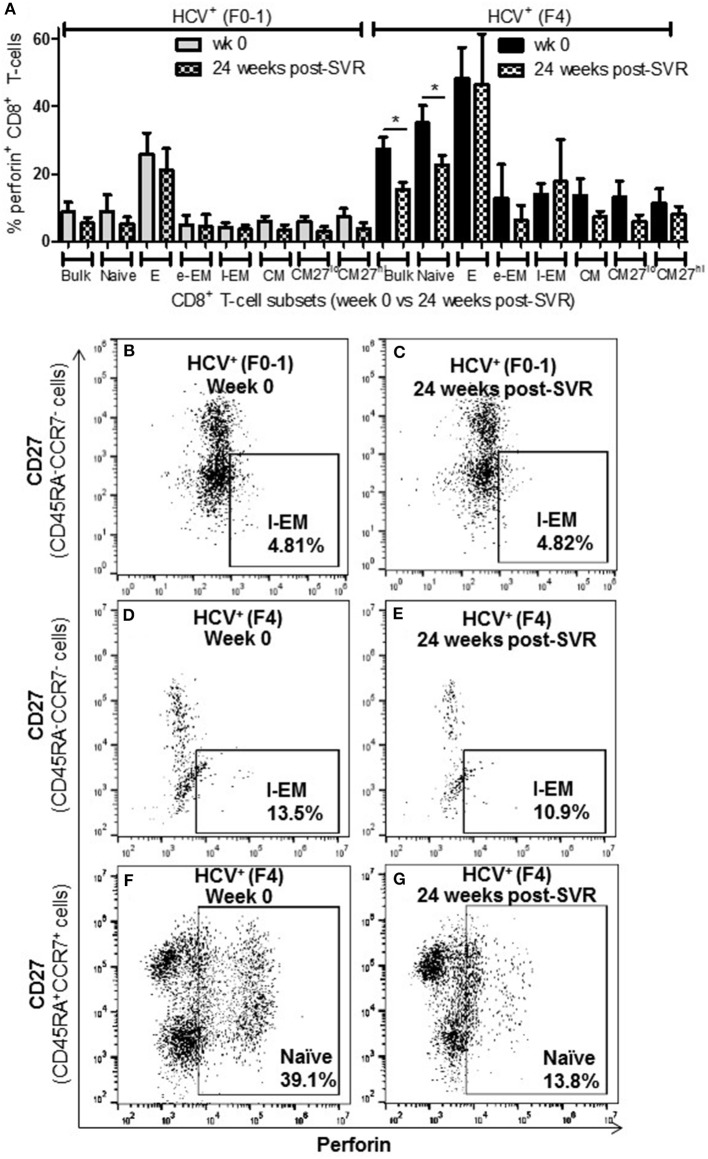
The proportion of perforin^+^ CD8^+^ T-cells remains elevated in most cell subsets long after elimination of chronic HCV infection in individuals with advanced liver fibrosis. Isolated CD8^+^ T-cells from PBMCs collected from HCV-infected individuals before treatment with direct-acting anti-HCV therapy (DAA) and 24 weeks post-SVR were stimulated with anti-CD3/–CD28 for 48 h followed by the detection of perforin^+^ cells were detected in cell subsets by flow cytometry. **(A)** The proportion of perforin^+^ cells in CD8^+^ T-cells and subsets thereof is summarized in a bar graph, showing data for week 0 and 24 weeks post-SVR in colored and hatch bars, respectively. Statistical significance is indicated with a “^*^” (one-way ANOVA and Dunnett's post-test, *p* ≤ 0.05, *n* = 4 in each study group). Representative dot plots demonstrate **(B,C)** the low level of perforin^+^ l-EM cells in HCV-infected individuals with minimal fibrosis, and how this was stable long after therapy compared to the higher proportion of perforin^+^ l-EM cells in individuals with advanced liver fibrosis **(D,E)**. **(F,G)** Finally, a representative dot plot depicts how the increased proportion of perforin^+^ naïve CD8^+^ T-cells in a sample from an HCV-infected individual with advanced liver fibrosis was the only subset to demonstrate a significant decrease in perforin expression after DAA therapy.

Nearly 1 year after the initiation of DAA therapy (i.e., 24 weeks post-SVR), there was a clear pattern of sustained CD8^+^ T-cell activity in HCV^+^(F4) individuals (Figures 5A, 6A, 7A). In this study group, the different cell functions were stable in many cell subsets after DAA therapy as seen by stable group means from week 0 to 24 weeks post-SVR (for representative examples, see Figures 5D vs. E, Figures 6D vs. E, Figures 7D vs. E). The exception to the observations of sustained cell function after DAA therapy, was the reduced proportion of perforin^+^ CD8^+^ T-cells (group mean: wk 0 = 27.39%, 24 weeks post-SVR = 15.25%, *p* = 0.01, Figure 7A). This was reflected by the only subset-specific reduction in perforin expression, observed in naïve cells (Figures 7F vs. G, group mean: wk 0 = 34.99%, 24 weeks post-SVR = 22.60%, *p* = 0.04). Therefore, the clearance of HCV by DAA therapy had a significant effect on CD8^+^ T-cell functions, particularly in HCV^+^(F4) individuals, whose exaggerated level of CD8^+^ T-cell activity was retained despite viral clearance with therapy. Metavir scores of liver fibrosis did not change significantly in either study group by 24 weeks post-SVR, therefore liver fibrosis reversal during and after therapy could not be associated with these observations. This concurs with our recent study that found that DAA therapy did not significantly decrease liver thickness (ave. 9.7 kPa at week 0 vs. 7.5kPa 24 weeks post-SVR, *n* = 23) (26). Furthermore, fibrosis scores of F(4) individuals did not decrease with statistical significance and remained classified as F4 (*n* = 5, week 0 ave. 21.6 kPa vs. 24-weeks post-SVR ave. 14.6 kpa). The fibrosis measures of F(0-1) individuals also did not decrease significantly in this study (*n* = 9, week 0 ave. 4.9 kPa vs. 24-weeks post-SVR ave. 5.0 kPa). Treatment with DAA did not alter cell subset distribution by 24 weeks SVR in any of the study groups (data not shown). In addition, we did not detect a different in CMV serostatus among the DAA-treated individuals studied. However, nearly all individuals were CMV-antibody positive (7/8, see Table 1).

### Plasma Cytokine Concentrations Before and After DAA Therapy Differ Between Individuals With Minimal or Advanced Liver Fibrosis

To complement our studies of circulating CD8^+^ T-cell function, the concentrations of pro- and anti-inflammatory cytokines were quantified in the plasma of HCV-infected individuals across a spectrum of liver fibrosis severities (F0-1 *n* = 8, F4 *n* = 4), before and after DAA therapy. Using multiplexed immunobead assays, several pro- and anti-inflammatory cytokines, chemokines and growth factors were significantly increased across the all HCV^+^ individuals before therapy, compared to healthy controls (*n* = 4) (see Table 2). Despite the small sample size and individual variation in plasma cytokine concentrations, several distinct differences were found when analyzing HCV^+^ individuals based on the severity of liver disease. At baseline, the (F4) group expressed significantly higher concentrations of pro-inflammatory tumor necrosis factor-related apoptosis-inducing ligand (TRAIL) and chemokine MIG (monokine-induced by gamma-IFN, CXCL9) and tended to express more IL-8 compared to the (F0-1) group. There was a particularly significant difference in plasma TGF-β concentrations between the groups. The (F4) group expressed significantly less anti-inflammatory TGF-β. A previous report found similar levels of plasma TGF-β in HCV^+^ individuals of which 80% had F4 fibrosis, and these concentrations were significantly lower than that of healthy controls (ave. 3,800 pg/ml) (31). Other growth factor differences in the (F4) group included decreased G-CSF and increased HGF concentrations compared to the (F0-1) group.

**Table 2:** Plasma cytokine concentrations in HCV infected individuals with minimal or advanced liver fibrosis before and after direct-acting antiviral therapy.

	**Baseline**					**24 weeks post-SVR**			
	Control^a^ (*n* = 4)	All HCV^+^ vs. Ctl (*P*-value)^b^	HCV^+^ (F0-1) (*n* = 8)	HCV^+^ (F4) (*n* = 4)	HCV^+^ F0-1 vs. F4 (*P-*value)	HCV^+^ (F0-1) 24 weeks post-SVR	F0-1 Δrel.^f^ to baseline (*P-*value)	HCV^+^(F4) 24 weeks post-SVR	F4: Δrel. to baseline (*P-*value)
**Pro-inflammatory**									
*IFN-y*	BDL		0 (0–4)^c^	0 (0–1)		BDL		BDL	
IFN-a2	BDL		1.9 (1–12)	0 (0–1)		0.6 (0–3)	0.071	BDL	
IL-12p40	5.8		14.9 (8–45)	11.4 (6–35)		5.7 (3–267)		12.1 (6–27)	
IL-12p70	BDL		3.5 (2–23)	BDL		BDL	**0.030**	BDL	
IL-18	2.6	**0.0003, +**	23.4 (15–59)	60.2 (37–71)		15.0 (8–40)	**0.004**	22.9 (21–33)	
IL-2Rα	0.5	**0.001, +**	57.6 (45–72)	63.7 (56–72)		33.3 (21–44)	**0.004**	47.3 (31–56)	
IL-6	BDL	**0.053, +**	0.7 (1–4)	1.4 (1–2)		BDL	**0.053**	BDL	
IL-8	0.3	**0.001, +**	8.0 (5–11)	13.2 (9–21)	0.064	5.8 (4–14)		9.2 (6–22)	
TNF-α	BDL	**0.034, +**	3.4 (2–82)	2.3 (1–3)		BDL	**0.018**	2.3 (1–3)	
TNF-β	1.3		7.4 (4–35)	1.2 (1–2)		2 (1–131)		0.7 (0–1)	
TRAIL	18.0		23.3 (15–30)	7.2 (4–17)	**0.033**	24.4 (12–30)		2.6 (1–10)	
**Anti-inflammatory**									
IL-1Rα	19.6	**0.002, +**	171.4 (108–326)	93.2 (18–161)		150.6 (82–263)		115.1 (79–151)	
IL-10	BDL		0.8 (0–7)	BDL		0.6 (0–7)	**0.500**	BDL	
IL-9	22.9	**0.047, +**	65.8 (43–162)	37.7 (31–47)		36.7 (26–268)		32.6 (27–37)	
MIF	86.7	**0.011, +**	171.2 (110–406)	420 (223–566)		276.2 (16–550)		416.5 (262–478)	
TGF-β^d^		na^e^	6373 (4908–25595)	3283 (2963–4848)	**0.010**	5430 (2863–20165)	**0.001**	2154 (1740–3078)	**0.03**
**Chemokines**									
CTACK (CCL27)	7.6	**0.002, +**	115.6 (66–174)	145.9 (112–		44.3 (23–92)	**0.020**	115.1 (61–143)	
Eotaxin	19.2	**0.002, +**	433.6 (225–486)	283 (153–363)		286 (147–361)	**0.012**	204.9 (111–306)	
GRO–α (CXCL1)	29.8		55.7 (28–87)	71.3 (68–75)		0 (0–19)	**0.011**	24.5 (12–44)	0.063
IP-10 (CXCL10)	36.3	**0.0001, +**	1896 (1087–3191)	2040 (1684–3053)		257 (111–347)	**0.004**	366 (284–528)	0.063
MCP-1 (CCL2	2.3	**0.001, +**	35.3 (28–56)	34.7 (19–63)		32.9 (20–37)		26.5 (15–48)	
MIG (CXCL9)	585.1	**0.026, +**	1209.5 (1116–1470)	2794 (2282–3358)	**0.001**	688.4 (449–693)	0.074	1482 (900–1849)	0.063
MIP-1α (CCL3)	0.6	**0.031, +**	1.6 (1–2)	2.3 (2–3)		0.9 (0–2)	**0.004**	1.4 (1–2)	0.063
MIP-1β (CCL4)	20.0	**0.003, +**	41.6 (34–53)	47.2 (38–48)		31.8 (27–51)	**0.054**	32.3 (31–36)	
RANTES (CCL5)	2420.7	**0.013, +**	5845.5 (4882–7406)	7960 (4694–10224)		6104 (5247–7556)		8460 (5943–8758)	
**Growth factors**									
G-CSF	4.3	**0.007, +**	57.2 (38–129)	17.4 (13–31)	**0.015**	26.3 (15–62)		12.8 (6–19)	
HGF	117.3	**0.019, +**	192.9 (144–217)	243 (232–481)	**0.010**	160.0 (97–237)		236.4 (216–511)	
IL-7	BDL		BDL	BDL		BDL		BDL	
LIF	BDL	0.064, +	8.1 (4–68)	BDL		0.8 (0–5)	**0.047**	BDL	
M-CSF	BDL	**0.001, +**	20.0 (16–26)	21.7 (16–24)		7.1 (4–11)	**0.004**	10.6 (6–13)	

a *Control sample concentrations reported as means and IQR*.

b *Statistical significance was determined by Mann-Whitney U-Test (p ≤ 0.05) and are highlighted in bold, and statistical trends are included (p = 0.06–0.08)*.

c *Median and interquartile ranges (IQR) are rounded to the nearest whole number*.

d *TGF-β concentrations were determined in a separate assay*.

e *n/a, data not available*.

f *Δ relative change compared to baseline (i.e., week 0, pre-DAA therapy)*.

After DAA therapy (i.e., 24 weeks post-SVR), the concentrations of several cytokines decreased significantly in HCV^+^(F0-1) individuals compared to baseline (Table 2). Some cytokines underwent small and unlikely biologically relevant changes after treatment, and the small sample size and individual variation in marker concentrations confounded the interpretation of treatment or group effects. However, significant reductions in the concentrations of key pro-inflammatory cytokines (IL-18, IL-2Rα, IL-8 and TNF-α) and several chemokines were observed. The elevated levels of TGF-β1 at baseline in the HCV^+^(F0-1) group decreased significantly at the end of treatment (*p* = 0.01, week 12, data not shown) as reported previously (32), and this was maintained up to 24 weeks post-SVR. In contrast, most plasma cytokine concentrations remained unchanged in the HCV^+^(F4) group, with trends toward decreases in chemokines Gro-α (CXCL1), IP-10 (CXCL10) MIG, and MIP-1α (CCL3). Plasma TGF-β1 levels remained stable throughout week 12 and then further decreased by 40% at 24 weeks post-SVR.

## Discussion

Immune dysfunction in advanced liver disease has been described previously and includes a disruption of normal T-cell activity. In chronic HCV infection, the impairment of virus-specific CD8^+^ T-cells has been widely documented yet the broader effects on circulating CD8^+^ T-cells is less well-understood. We observed dramatic differences in the phenotypic distribution of CD8^+^ T-cell subsets in blood between untreated HCV^+^(F0-1) and HCV^+^(F4) individuals. We also observed hyperfunctional activity of several CD8^+^ T-cell subsets, in a generalized manner, in HCV^+^(F4) individuals. In contrast, the function of bulk CD8^+^ T-cells in HCV^+^(F0-1) individuals were typically similar to that of uninfected healthy controls. The generalized hyperfunction of circulating CD8^+^ T-cells from HCV^+^(F4) individuals was largely sustained up to a year post-treatment initiation, particularly with perforin production and cellular cytotoxicity. It is possible that this hyperfunction is sustained in part by the ongoing systemic inflammation and reduced anti-inflammatory signaling after HCV cure. This is the first demonstration that DAA therapy does not resolve systemic immune cell changes in HCV^+^ individuals with advanced liver disease.

Differences in CD8^+^ T-cell subset distribution in treatment-naïve HCV-infected individuals with disparate degrees of liver fibrosis suggested there may be also functional differences. Our previous work had shown significant reductions in naïve CD8^+^ T-cells in chronic HCV infection using a cell phenotyping method involving CD45RA and CCR7 cell surface markers but did not detect differences between individuals with minimal vs. advanced liver fibrosis (*n* = 8 vs. *n* = 4, respectively) (21). In the present study, the inclusion of the co-stimulatory receptor CD27 as a marker in our cell subset determination strategy is arguably more precise in its distinction between effector and memory cell subsets (28, 30). We detected profound decreases in the proportion of naïve cells in the HCV^+^(F4) group and a significant over-representation of effector and late effector memory cells. In untreated HCV^+^(F4), the low number of naïve cells may be due to the expansion of effector and memory cell compartments (particularly l-EM cells) compared to that of HCV^+^(F0-1) individuals or healthy controls. We also observed that the decreased proportion of naïve CD8^+^ T-cells was sustained long after SVR with DAA therapy, as reported by others (33). It has been suggested that reduced expression of CD5, a negative regulator of T-cell receptor signaling, may be associated with such sensitivity to activation in naïve T-cells (33). Rapid and sustained increases in CD4^+^ and CD8^+^ T-cell numbers during DAA treatment with immediate reductions in viral load may reflect an efflux of hepatic lymphocytes from the liver as the virus is cleared and liver inflammation reduces (34, 35). The functional consequence has not been addressed, yet the increased proportion of cells expressing markers of immune activation (e.g., CD38^+^HLA-DR^+^) in HCV^+^(F4) individuals may reflect these differences. Human naïve and memory CD8^+^ T-cells have recently been shown to differ fundamentally in their metabolic programming upon activation (36), and the effects of chronic disease on cell proportions may be an indicator of other underlying biochemical differences.

The degree of T-cell activity that we observed in HCV^+^(F4) individuals suggests an altered threshold for T-cell functionality, compared to HCV^+^(F0-1) individuals or healthy controls. Immune activation with aberrant activation of CD4^+^ and CD8^+^ T-cells is a hallmark of untreated HIV mono- and HIV-HCV co-infection (37) and Barrett et al., have suggested that their findings with regards to strong HCV-specific T-cell responses are related to liver fibrosis severity (38). The antigen specificities of these cells are largely unknown hence either antigen-dependent or -independent mechanisms resulting from an inflammatory milieu and microbial translocation may be the underlying cause (39). This bystander effect is thought to skew memory/effector cell differentiation toward exhaustion, marked by high levels of PD-1 (40, 41). Increased numbers of IFN-γ^+^ naïve cells may seem like a curious finding (Figure 2) yet has been previously reported along with increased granzyme B expression yet was not stratified by the degree of liver fibrosis (33). The latter study found that these hyperfunctional naïve cells recovered >2 yrs post-SVR after IFN-α + ribavirin (*n* = 5) or IFN-α-free (*n* = 2) therapies. Our data indicates that this recovery is not so evident after DAA therapy in HCV^+^(F4) individuals and may have important repercussions if altering naïve cells, thereby influencing T-cell pre-immune repertoire with clinical outcomes that remain to be identified. Elevated proportions of perforin^+^ naïve cells may further support this possibility (Figure 4A). Perhaps the generalized state of hyperfunctional CD8^+^ T-cells in HCV^+^(F4) individuals will weaken their responsiveness to antigen-specific stimuli, resulting in reducing the efficacy of immune responses while contributing to bystander tissue damage. Tissue damage may be a possible outcome of elevated perforin^+^ EM cells, which may gain access to peripheral tissues including the liver by virtue of tissue homing receptors. The phenomenon of bystander activation of T-cells, including that of naïve cells, is increasingly noted for having a role in exacerbating disease severity (42).

The association of CD8^+^ T-cell hyperfunction in chronic HCV infection with immune activation is not well-understood and is in contrast to the reports on the impairment of *HCV-specific* CD8^+^ T-cells in circulation and in the liver (11–16). While an evaluation of antigen-specific responses to HCV may have added some insights here, limited numbers of such cells in circulation is a significant technical challenge, as previously noted (38). The responses to anti-CD3/-CD28 stimulation measured here may not represent responses to HCV infection. In this post-cure era, our rationale was to gain perspectives of host response that extend beyond HCV-specific responses, which have been reported on extensively and it is well-accepted that those responses are impaired and contribute to the inability to clear HCV infection. Rather, our findings will inform the potential of the HCV-non-specific CD8^+^ T-cell population as it pertains to remaining clinical issues such as future responses to other infections, routine vaccination and cancers. Viral factors of HCV have been associated with immune impairment, including our recent demonstration of how HCV core protein can impair CD8^+^ T-cell activities (29). Whether host factors associated with liver fibrosis severity determine the degree of CD8^+^ T-cell function and immune restoration potential after DAA treatment has not been addressed until our study. Age-related effects on immune function are known, yet the two HCV^+^ groups studied here did not differ significantly by age (F0-1: ave 57.3 ± 7.8 (range 41–64), F4: ave 59.9 ± 11.7 (range 45–76). A stepwise loss of CD8^+^ T-cell functions is possible, as in models of chronic viral infection (43), wherein retention of some activities occurs at the expense of others as one progresses toward decompensated cirrhosis. Recent studies described hyperfunctional circulating CMV-/EBV-specific CD8^+^ T-cells (increased intracellular expression and release of IFN-γ and TNF-α or detectable CD107a expression) in chronic HCV infection, despite elevated expression levels of exhaustion markers PD-1, Tim-3 and 2B4 (44). While DAA therapy partially restored CD8^+^ T-cell exhaustion marker expression by the time of SVR, cellular functions remained elevated, suggesting a disconnect between regulatory and activating signals. Nearly all of the DAA-treated subjects in our study were CMV-seropositive (7/8, Table 1), preventing any assessment of CMV reactivation effects on CD8^+^ T-cells known to be associated with chronic viral infection and aging (45). Our data, and that of our previous work, showed little change in the activity of the l-EM subset (21) whose function is typically enhanced with CMV reactivation (46, 47). Collectively, such immune cell activity may be a feature of bystander expansion mediated by cytokines or other cell-cell contact events, either in the liver or in circulation, and may not reflect the dysfunction widely documented in HCV-specific CD8^+^ T-cells.

Extreme immune dysfunction in liver disease (cirrhosis-associated immune dysfunction syndrome) includes transition through states of immune depression/exhaustion and overstimulation/activation, depending on the liver disease severity and etiology (1). Whether our findings are related to the effects of chronic HCV infection or progressive liver disease, or a combination thereof, remains to be determined through studies in cirrhotic HCV uninfected individuals. The mechanisms underlying CD8^+^ T-cell dysfunction in this context are not known. Hyperfunctional CD8^+^ T-cells may be predisposed to activation-induced cell death (33). We have previously observed systemic and local effects on liver CD8^+^ T-cells in HCV infection (21). We specifically found that CD8^+^ T-cells from HCV^+^(F4) individuals produce less anti-apoptotic Bcl-2 in response to the survival cytokine IL-7 compared to cells from HCV^+^(F0-1) individuals. Since the liver filters one third of the body's blood volume every minute, circulating cells can interact briefly yet regularly with the molecular and cellular microenvironment of the increasingly damaged and inflamed organ (48). The liver is an important source of the cytokine IL-7 (produced by hepatocytes) which mediates T-cell survival as well as CD8^+^ T-cell cytotoxic activity as determined in a murine model (49). Accumulating liver damage therefore reduces this source of cytokine for circulating T-cells which we have found to express unchanged levels of the IL-7 receptor α chain (21), a situation which may persist if liver fibrosis regression does not occur after anti-HCV therapy. Interestingly, immune blockade inhibitors ±IL-7 *in vitro* failed to reverse the dysfunction of HCV/CMV/EBV-specific CD8^+^ T-cells from HCV-infected individuals with rapid fibrosis progression (50). Since we did not observe any reversal in liver fibrosis scores during or after therapy, we could not test whether liver fibrosis regression influenced T-cell activity.

The results of this study expose an interesting relationship between generalized CD8^+^ T-cell activity and liver fibrosis severity. A limitation to the interpretation of these findings is that the assessment of function after 48 h of *in vitro* stimulation may not accurately reflect the status of cells *in vivo* in contrast to a 6 h stimulation (Figure S1). In addition, sample size posed a major limitation to data interpretation, with some responses fraught with individual variation in function which may have been alleviated with additional patient sampling. Until recently, access to funding for DAA therapy was extremely limited in our province, therefore limiting access to study samples, as reflected in several reports with similar sample sizes in this field around the world (20, 51, 52), while others have managed larger data sets as DAA treatment became more accessible [e.g., Owusu Sekyere et al. (44)]. However, many of these studies had low proportions of cirrhotic HCV^+^ patients, further highlighting difficulties in reaching ideal sample sizes [e.g., Alanio et al. (33)]. Despite this, we are confident that our findings are compelling and novel with regards to linking hyperfunction of T-cells to advanced liver fibrosis. Efforts are ongoing to overcome such important sample size limitations to cell-based research. We are increasing our capacity to collect PBMC from HCV-infected individuals undergoing DAA therapy, including long-term follow up, and storing samples in our new local biobank (now supported in part by the Canadian Network for Hepatitis C) for ongoing research.

Lasting T-cell dysfunction in HCV^+^(F4) individuals is of great clinical significance, although studies on this have been limited. The enduring effects observed in our study complement a previous report of how one HCV-specific CD8^+^ T-cell subset is maintained after DAA therapy, while other exhausted cells are not (53). Irreversible immune cell dysfunction has been noted following long-term IFN-α therapy (48 weeks) (24), suggesting that it is not an observation restricted to the use of DAA therapy. Collectively, our data and these reports suggest that a chronic viral infection and advanced liver disease may underlie an imprinting of immune cells resulting in long-term dysfunction, even after viral cure. Effects on heterologous immunity should also be considered (54). There is an increased risk of community-acquired infections such as pneumonia in cirrhosis (55, 56) and poor responses to influenza (57, 58) or hepatitis B (59–61) vaccines by HCV-infected individuals, all of which rely on effective antibody responses. Our understanding of the effects on *de novo* or recall CD8^+^ T-cell responses is less well-understood. Ongoing systemic inflammation after DAA therapy has been associated with sustained CD8^+^ T-cell dysfunction (23), although we can only speculate that systemic inflammation may contribute to observed differences in CD8^+^ T-cell functions in our study groups. We observed sustained elevated levels of plasma TGFβ in HCV^+^(F4) individuals (Table 1). Epigenetic imprinting of TGFβ has recently been associated with hyperfunctional NK cells (increased IFN-γ and TNF-α production) (62), so this mechanism may extend to T-cells and should be investigated further. Since functional HCV-specific memory CD8^+^ T-cells are absolutely required for protection from HCV reinfection (63), HCV vaccines currently under development must emulate effective T-cell responses. Alterations to naïve cells may also influence the potential for inducing *de novo* T-cell memory to other viral antigen epitopes. Our data suggest that lasting CD8^+^ T-cell dysfunction after HCV cure in HCV^+^(F4) individuals could result in a failure to generate effective responses to such a vaccine when it becomes available, as others have predicted (64).

Lastly, the risk of developing liver cancer increases dramatically with progressive liver fibrosis in HCV^+^ individuals. While DAA therapy is thought to reduce the risk of HCC attributed to HCV infection (8, 9), two recent yet controversial studies suggest there may be an increased HCC recurrence (≈30%) after DAA therapy, whereas IFN therapy was only associated with 1–2% recurrence rates (65, 66). This recurrence is thought to be mediated in part by a derangement of immune surveillance function of CD8^+^ T-cells, due to the rapid decrease in viral load with DAA and the reduction of the IFN response causing an imbalanced anti-tumor response (67). Determining the underlying mechanisms of sustained CD8^+^ T-cell dysfunction after DAA therapy in HCV^+^(F4) individuals will identify novel therapeutic add-ons to improve clinical outcomes, particularly in this era of rising rates of HCC.

In conclusion, our association of liver fibrosis severity to CD8^+^ T-cell activity in chronic HCV infection highlights a potentially important correlate of systemic immune dysfunction. The sustained hyperfunction of CD8^+^ T-cells long after DAA therapy suggests how profoundly affected the immune system is by an HCV infection and advanced liver disease. The mechanism by which this occurs is not well-understood, and the search for immune-restoring interventions is an active area of research (68). The long-term consequences for altering T-cell response thresholds with advanced liver disease are not known but may be relevant in pathogenic situations in the context of chronic HCV infection or after HCV cure. Remaining clinical sequalae post-cure, in which CD8^+^ T-cell play a prominent role, include new, and often more aggressive forms of the HCC (69), or an increased HCC recurrence (65) and extrahepatic cancers (70). There is concern that individuals who experienced chronic HCV infection may fail to generate effective HCV vaccine responses when available, as they will require *de novo* T-cell activity, posing a challenge to vulnerable populations where HCV re-infection is a risk (64). Hyperfunctional CD8^+^ T-cells may be a double-edged sword with beneficial robust immune responses to certain infections or ineffective, overactive and tissue-damaging cytotoxic activity. Several important studies have identified lasting epigenetic changes in the mouse model of chronic LCMV infection and in small studies of either HIV of HCV infection (71). Whether a state of advanced liver fibrosis further contributes to this remains to be determined. Understanding the mechanisms of immune dysfunction and barriers to immune restoration after HCV cure will aid in mitigating the remaining negative long-term health outcomes for individuals with advanced liver fibrosis.

## Ethics Statement

This study was carried out in accordance with the recommendations of the guidelines established by the Ottawa Health Science Network Research Ethics Board with written informed consent from all subjects. All subjects gave written informed consent in accordance with the Declaration of Helsinki. The protocol was approved by the Ottawa Health Science Network Research Ethics Board.

## Author Contributions

AV, FD, and SK developed the immunoassays and performed the experiments. AV, SK, and AC graphed and analyzed the data. JA aided in the analysis of data and edited the manuscript. CC aided in the design of the study, facilitated the acquisition of study samples, and edited the manuscript. AC designed the study and wrote the manuscript.

### Conflict of Interest Statement

The authors declare that the research was conducted in the absence of any commercial or financial relationships that could be construed as a potential conflict of interest.

## Supplementary Material

Figure S1 Dose response and time course analysis of anti-CD3/-CD28 stimulation of isolated CD8^+^ T-cells. To determine the appropriate dose of stimulation reagents and culture time to detect robust functional readouts in bulk CD8^+^ T-cell cultures, time course and dose response experiments were conducted. Representative graphs of such experiments are shown here. **(A)** First, the concentration of pre-coated anti-CD3 and soluble anti-CD28 antibodies was titrated (1–20 μg/ml) was evaluated across a time period of 6–72 h. The proportion of IFN-γ^+^ cells was measured by flow cytometry, highlighting the significant detection of IFN-γ^+^ cells at 48 h. Additional experiments over similar time courses were conducted with 10 μg/ml of anti-CD3/-CD28 antibodies, measuring **(B)** CD107a^+^ and **(C)** perforin^+^ CD8^+^ T-cells.

Figure S2 The effect of anti-CD3/-CD28 stimulation *in vitro* on CD8+ T-cell subset distribution. CD8^+^ T-cells isolated from PBMCs were stimulated with anti-CD3/CD28 antibodies (10 μg/ml) for 48 h followed by an evaluation of phenotype distribution, alongside function assessment. The proportions of cell CD8^+^ T-cell subsets were distinguished based on surface marker expression by flow cytometry as follows: Naïve (CD45RA^+^CCR7^+^CD27^+/−^), Effector (E, CD45RA^−^CCR7^−^CD27^−^), Early Effector Memory (e-EM, CD45RA^−^CCR7^−^CD27^+^), Late Effector Memory (l-EM, CD45RA^+/−^CCR7^−^CD27^−^) and Central Memory (CM, CD45RA^−^CCR7^+^CD27^+/−^). The distribution of subsets in **(A)** uninfected controls (*n* = 9) and treatment naïve HCV^+^ individuals with **(B)** minimal (Metavir score F0-1, liver thickness ≤7.0 kPa, *n* = 9) or **(C)** advanced liver fibrosis/cirrhosis (F4, ≥12.5 kPa, *n* = 5) are shown in bar graphs (error bars represent SD). Unstimulated cells are shown in clear bars, whereas stimulated cells are shown with gray bars. Statistically significant changes in subset proportions with stimulation compared to unstimulated controls within each subset were determined by two-way, paired Student's *t*-tests and indicated with an asterisk “^*^” (*p* ≤ 0.05).

## Abbreviations

CFSE: carboxyfluorescein succinimidyl ester
CMV: cytomegalovirus
CM: central memory
DAA: direct-acting antiviral therapy
EM: effector memory
e-EM: early effector memory
EBV: Epstein-Barr virus
F0-1: Metavir score for minimal liver fibrosis
F4: Metavir score for advanced liver fibrosis
HCV: hepatitis C virus
l-EM: late effector memory
MLR: mixed lymphocyte reaction
SVR: sustained virological response.
