# Supplementary figures and images for: Direct-Acting Antiviral Treatment of HCV Infection Does Not Resolve the Dysfunction of Circulating CD8+ T-Cells in Advanced Liver Disease
Source: Front Immunol. 2019 Aug 13;10:1926. doi: 10.3389/fimmu.2019.01926 (PMC6700371; doi:10.3389/fimmu.2019.01926)

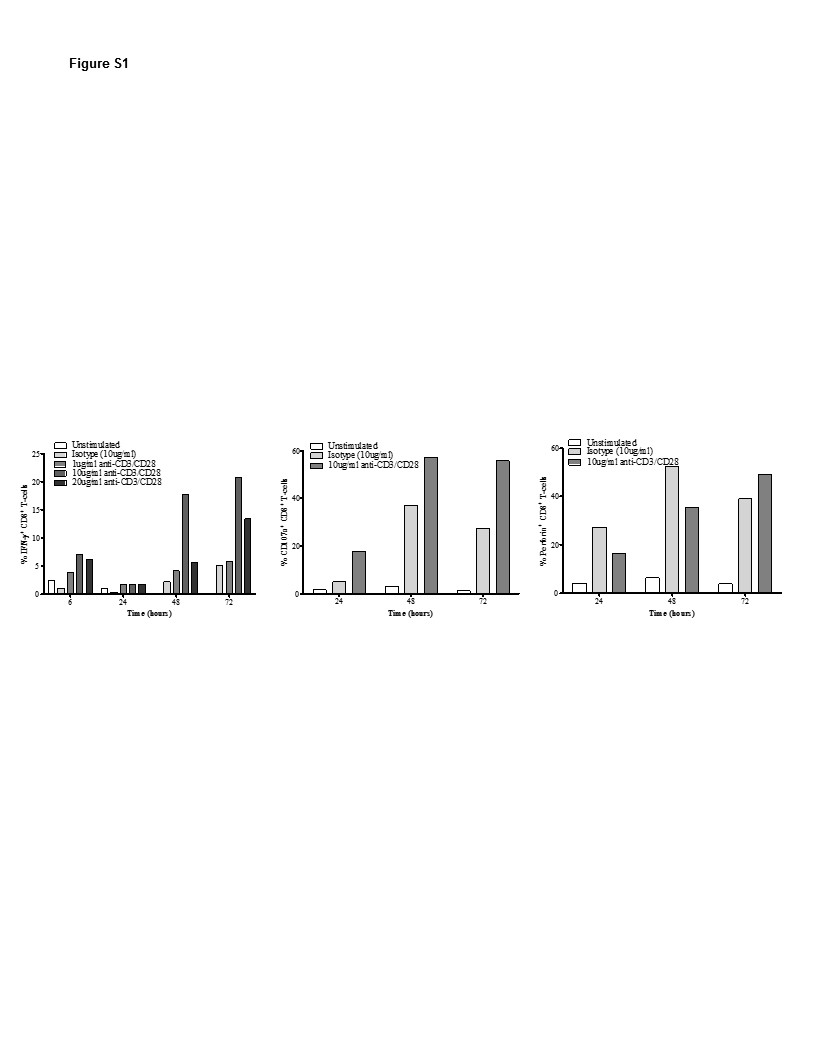

Supplement: Figure S1 — Dose response and time course analysis of anti-CD3/-CD28 stimulation of isolated CD8+ T-cells. To determine the appropriate dose of stimulation reagents and culture time to detect robust functional readouts in bulk CD8+ T-cell cultures, time course and dose response experiments were conducted. Representative graphs of such experiments are shown here. (A) First, the concentration of pre-coated anti-CD3 and soluble anti-CD28 antibodies was titrated (1–20 μg/ml) was evaluated across a time period of 6–72 h. The proportion of IFN-γ+ cells was measured by flow cytometry, highlighting the significant detection of IFN-γ+ cells at 48 h. Additional experiments over similar time courses were conducted with 10 μg/ml of anti-CD3/-CD28 antibodies, measuring (B) CD107a+ and (C) perforin+ CD8+ T-cells. [file Image_1.JPEG]

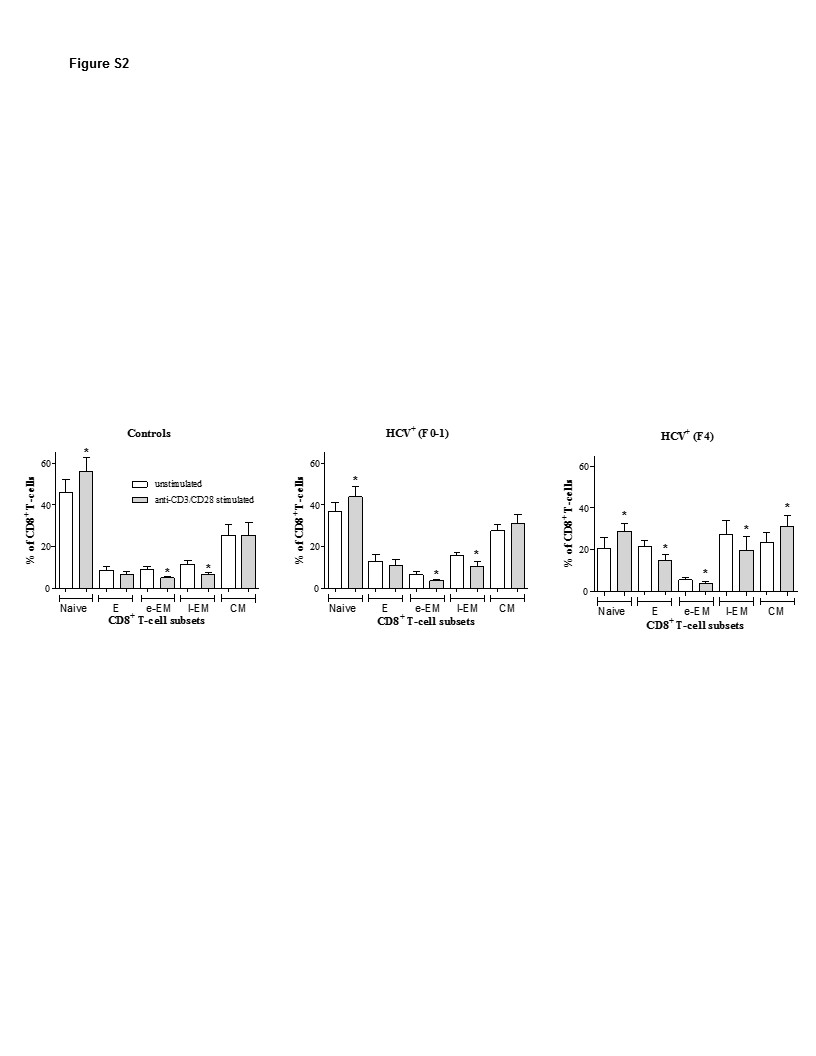

Supplement: Figure S2 — The effect of anti-CD3/-CD28 stimulation in vitro on CD8+ T-cell subset distribution. CD8+ T-cells isolated from PBMCs were stimulated with anti-CD3/CD28 antibodies (10 μg/ml) for 48 h followed by an evaluation of phenotype distribution, alongside function assessment. The proportions of cell CD8+ T-cell subsets were distinguished based on surface marker expression by flow cytometry as follows: Naïve (CD45RA+CCR7+CD27+/−), Effector (E, CD45RA−CCR7−CD27−), Early Effector Memory (e-EM, CD45RA−CCR7−CD27+), Late Effector Memory (l-EM, CD45RA+/−CCR7−CD27−) and Central Memory (CM, CD45RA−CCR7+CD27+/−). The distribution of subsets in (A) uninfected controls (n = 9) and treatment naïve HCV+ individuals with (B) minimal (Metavir score F0-1, liver thickness ≤7.0 kPa, n = 9) or (C) advanced liver fibrosis/cirrhosis (F4, ≥12.5 kPa, n = 5) are shown in bar graphs (error bars represent SD). Unstimulated cells are shown in clear bars, whereas stimulated cells are shown with gray bars. Statistically significant changes in subset proportions with stimulation compared to unstimulated controls within each subset were determined by two-way, paired Student's t-tests and indicated with an asterisk “*” (p ≤ 0.05). [file Image_2.JPEG]
